# Ocular Manifestations of Spondyloarthritis

**DOI:** 10.31138/mjr.34.1.24

**Published:** 2023-03-31

**Authors:** Akshat Pandey, Vinod Ravindran

**Affiliations:** 1 Apollo Hospitals, Indore, MP, India,; 2 Centre for Rheumatology, Calicut, Kerala, India and Department of Medicine, Kasturba Medical College, Manipal, India

**Keywords:** ankylosing spondylitis, uveitis, spondyloarthritis, eye manifestations, ocular, arthritis

## Abstract

**Introduction:**

Ocular manifestations are seen in several autoimmune disorders including spondyloarthritis (SpAs). Though acute anterior uveitis (AAU) is the hallmark of SpAs, episcleritis and scleritis are also seen. Genetic and geographical factors impact the prevalence of AAU; however, available evidence support that HLA-B27 positivity is closely associated with it.

**Objective:**

The current narrative review is focused on clinical features of AAU and its management.

**Methods:**

For the purpose of this narrative review; literature search covered MEDLINE, Google scholar and EMBASE databases and included articles published in English language from January 1980 till April 2022 using the following keywords: “Ankylosing spondylitis”, “Spondyloarthritis”, “Eye manifestations”, “Ocular”, “Uveitis” and “Arthritis”.

**Conclusion:**

Patients with SpA may suffer from different ocular complications with uveitis being the most common one. Biological therapy is a promising medical strategy enabling in achieving therapeutic goals with minimal adverse effects. An effective management strategy for patients with AAU associated with SpA could be formulated by the collaboration between ophthalmologists and rheumatologists.

## INTRODUCTION

Spondyloarthritis (SpA) is an umbrella term for a group of autoimmune rheumatic diseases comprising psoriatic arthritis, reactive arthritis, SpA related to inflammatory bowel diseases (IBD) and ankylosing spondylitis (AS) that affects the spine and peripheral joints. They have an estimated global incidence of 1%.^1^ Non-radiographic axial SpA is also common; a cross-sectional survey conducted in Asian patients with inflammatory back pain found that 36.46% of them met its classification criteria.^2^ The exact aetiopathogenesis of this condition is not yet fully understood; however, involvement of immune mediators on the background of several genetic, familial and environmental predispositions are considered important.

Autoimmune disorders such as SpAs exert pernicious systemic and ocular effects. Peripheral manifestations, especially inflammation of entheses (enthesitis) and fingers and toes (dactylitis) are the hallmark of SpA. Ocular problems are frequently encountered in SpA, of which uveitis is the hallmark which is reported in almost 40% of the patients. Uveitis can be infectious or non-infectious, and can be a part of either a systemic disease (rheumatological or otherwise) or a local process. Literature reports a higher recurrence rate, bilateral involvement and frequent posterior synechiae in patients with rheumatological disease associated uveitis than those without.^3^ The presentation of ocular manifestation varies in every individual ranging from mild to severe. Untreated uveitis increases the risk of further complications of chronic ocular inflammation resulting in blindness. Precise diagnosis with prompt treatment by ophthalmologist in collaboration with rheumatologist may mitigate the distressing ocular sequelae and complications. In a meta-analysis by Turk et al., type of ocular involvement in childhood and adult SpA was noted to be similar.^4^ However, occurrence of uveitis was less frequent in adult-onset PsA compared to child-onset PsA.^4^

In this narrative review we have appraised the various ocular manifestations of SpA; particularly Acute Anterior Uveitis (AAU) and present some perspectives on its management.

## SEARCH STRATEGY

For the purpose of this narrative review, literature search covered MEDLINE, Google scholar and EMBASE databases and included articles published in English language from January 1980 till April 2022 using the following keywords: “Ankylosing spondylitis”, “Spondyloarthritis”, “Eye manifestations”, “Ocular”, “Uveitis”, and “Arthritis”. **Table 1** lists some of the more relevant studies based on the search.

**Table 1.** Some of the important studies of uveitis associated with SpA.

**Study [Reference]**	**Study Design**	**Diagnosis/Number of patients**	**Study outcomes**
Vale et al., 2018^6^	Observational retrospective study	SpA/153	Uveitis predominantly occurred in men, and more frequently in HLA-B27 positive patients
Mitulescu et al., 2018^7^	Retrospective study	SpA/67	Patients with AAU and inflammatory back pain should be referred to a rheumatology unit.
Ninan et al., 2017^8^	Hospital based cross sectional	Seronegative /SpA	Uveitis percentage was 16.3%, AS was commonest occurring in 69.3% and 78.3% patients were HLA-B27 positive
Das et al., 2015^10^	Retrospective study	Uveitis of varying etiologies/343	Changing pattern of uveitis in a tertiary institute was reported with highest number of HLA-B27 associated uveitis.
Lee et al., 2017^11^	Retrospective, interventional case series	AS/91	Men preponderance (70%), average onset in the fourth decade, and unilateral manifestation (87.9%) was observed
Bouzid et al. 2020^31^	Retrospective study	SpA/101	First-line sulfasalazine reduced uveitis relapses
van Denderen et al., 2014^34^	Randomized controlled trial	AS/77	Adalimumab treatment. significantly reduced the number of acute uveitis attacks, and the number of attacks per patient
Rudwaleit et al., 2016^37^	Randomized, double-blind and placebo-controlled RAPID-axSpA (NCT01087762)	axial SpA/218	Certolizumab Pegol reduced uveitis flares
Calvo-Río et al., 2016^38^	Multi-center randomized trial	SpA/15	Golimumab is predicted to be useful therapeutic option in refractory SpA-related uveitis

## EPIDEMIOLOGY

The International Uveitis Study Group has classified uveitis according to the location, clinical course, and distribution.^5,6^ Depending upon the location of inflammation in tissue whether anterior or posterior to the lens it is labelled as anterior or posterior uveitis. Clinically it is classified by aetiology as infectious, non-infectious, and masquerade (heterogeneous group of eye diseases that mimic chronic intraocular inflammation).^5^ An unsatisfactorily extended delay of 5 to 10 years is generally observed among the first occurrence of SpA symptoms including uveitis and its diagnosis. This delay may be attributed to the lack of awareness regarding SpA and requirement of there being radiographic sacroiliitis grade 2 bilaterally or grade 3 or 4 unilaterally for its classification.

Acute anterior uveitis is the most common extra-articular manifestation in SpA which might be acute or chronic.^6,7,8^ It is the most frequent type of Uveitis prevalent globally though infectious causes are more common in the developing countries.^9^

The epidemiologic variations of uveitis depends on the type of SpA, disease duration and presence of HLA-B27 gene.^10^ Gender and ethnic variations are strong factors behind the epidemiological pattern seen. Male preponderance is observed with SpA associated uveitis with differences in clinical presentations in both genders; however, no definitive reason for these differences has been established as yet.^11,12^

From Europe an urban multi-ethnic population were investigated to explore the epidemiological spectrum of uveitis and reported 25% had immune associated uveitis.^13^ An observational survey in France which included 902 patients with SpA showed uveitis prevalence of 32.2% with recurrence rate of 52.3%.^14^ Factors independently associated with uveitis in this survey were noted to be the presence of HLA-B27 gene and disease duration for ≥ 10 years (P<0.0001).^14^ A systematic literature review has found an overall incidence of 32.7% of uveitis in patients with SpA with disease duration of 17.7±1.0 years.^15^

A strong co-relation exists between HLA-B27 gene and AAU; nevertheless, cases of undiagnosed SpA are common in patients with AAU, primarily in those who carry the HLA-B27 gene.^16^ Current evidence suggests that AAU linked with the HLA-B27 has a less favourable prognosis and prone to frequent relapses.^17^ In this context it is important to emphasise that the classification criteria proposed by the Assessment in Spondyloarthritis International Society (ASAS) of axial-SpA includes presence of HLA-B27 as a part of the clinical arm.^18^

## CLINICAL ASPECTS

Ocular involvement in SpA includes the sclera, episclera, the uveal tract, and other adjacent structures including vitreous humour, retina, optic nerve, and vessels. Uveitis in SpA indicates severity of the disease and can be bilateral too. Its characteristic feature is the “ping-pong” pattern meaning uveitis can affect alternatively one or the other eye.^5^

Blurred vision is the main symptom of AAU caused by ciliary spasm and presence of cells and/or flare in the anterior chamber of the eye; while pain, redness and watering are the other symptoms. Episodes of photophobia (due to ciliary muscle spasm secondary to inflammation in anterior chamber), circumcorneal injection (due to engorgement of episcleral vessels around ciliary body) and keratic precipitates, hypopyon (inflammatory cells in the anterior chamber of the eye), pupillary membrane (formed by the inflamed aqueous) and hyphema (blood cells in the anterior chamber) have also been reported.^9^

Persistence of uveitis leads to the development of posterior synechiae (adhesions between the lens and the iris). Secondary glaucoma can occur either as a part of the disease process or due to the excessive use of topical steroids. Other manifestations such as cataract and iris atrophy may also occur, though latter is the cardinal feature of herpetic anterior uvieitis.^5^

A multicentric Ibero-American cohort study demonstrated that 20% of patients with SpA had AAU, emphasizing that it is the commonest complication of SpA.^19^ Another finding from this study revealed that psoriatic arthritis was inversely related to AAU; however, it has been reported that uveitis is more deceptive in outbreak, chronic, posteriorly located and active bilaterally in PsA, when compared with other SpAs.^20^

Rudwaleit and colleagues compared patients with non-radiographic axial SpA and AS and did not find statistically significant differences in the prevalence of AAU, HLA-B27 and disease activity.^21^ Previous studies have shown that men are more prone to develop SpA-associated AAU, at an early age than women.^12,22^ However, rates of ocular manifestation and visual acuity were not predisposed to the gender.^12^

Another study from Taiwan has found that more than 90% of the patients with AS display the HLA-B27 positivity with the high proportion of the HLA-B*2704 subtype. It also reported an early disease onset and more relapses in HLA-B27-associated AAU.^23^ Although, HLA-B27 positivity is a key factor responsible for the incidence of SpA in patients with AAU, a wide range of incidence has been reported due to clinical and methodological differences including study design, classification criteria used for SpA, interobserver variability in radiography readings, and duration of follow-up.^24^

This variability of AAU among the different types of SpA was highlighted by several studies. Its higher prevalence in AS is perhaps a reflection of the higher frequency of HLA-B27-positive patients with AS.^22^ Among patients with psoriatic arthritis and IBD also a wide range of AAU prevalence associated with HLA-B27has been reported.^25^ Furthermore, a meta-analysis reported AAU as the most common (25.8%) extraarticular manifestation in patients with AS followed by psoriasis (9.3%) and IBD (6.8%).^26^

In ophthalmological settings, patients with AAU should be taken into consideration for the probability of a SpA diagnosis, especially in the presence of chronic back pain. Similarly, in rheumatological settings, presence of AAU in SpA is an useful indicator of a disease flare or suboptimal treatment. In accordance with the Assessment in Spondyloarthritis International Society/European League Against Rheumatism (ASAS-EULAR) recommendations for axial SpA, a rheumatologist can be the lead coordinator in a multidisciplinary network due to his/her thorough knowledge of the entire range of SpA.^27^ In this context, an algorithm called Dublin Uveitis Evaluation Tool (DUET) has been proposed to direct the ophthalmologists and physicians for timely referrals of patients with AAU to rheumatologists that will facilitate early detection of SpA.^28^

## MANAGEMENT

Early and accurate diagnosis for appropriate management of AAU is imperative to prevent morbidity and enhance the quality of life caused by poor eyesight and other complications. Management of AAU comprises local and systemic therapies depending on the gravity of problem and response to the treatment. The aetiology, anatomic site, type of onset, potential ophthalmic and systemic risks of drugs should be critically understood for accomplishing therapeutic success. The major goal of the treatment of SpA associated AAU is relief from acute attack and the prevention of relapses.

The treatment of uveitis is achieved through several strategies comprising of:

### Topical therapy

This is the standard treatment approach for symptomatic relief of an acute attack of AAU. A cycloplegic agent is used in combination with steroids that may be used systemically, topically, or by subconjunctival injection. Topical cycloplegics used with topical steroids prevents the formation of posterior synechiae.^29^ Furthermore, advances in the drug delivery systems has led to the development of intravitreal implants delivering steroids or other compounds. Systemic glucocorticoids (such as prednisolone or prednisone) are administered when topical treatment is ineffective and especially for bilateral uveitis.

### Systemic NSAIDs

Systemic NSAIDs inhibit prostaglandins and are used in mild conditions and inflammations non-responsive to topical therapy. They have been found to be an ideal alternative to glucocorticoids and are capable of reducing uveitis attacks.^29^

### Conventional synthetic disease-modifying antirheumatic drugs (csDMARDs)

The use of methotrexate in non-infectious uveitis has been found to decrease the rate of AAU flares during the withdrawal of systemic glucocorticoids.^30^ There is paucity in the data regarding treatment of AAU using sulfasalazine and leflunomide. A study by Bouzid *et al.* Has reported a reduction in uveitis relapses in a series of 101 patients with recurrent uveitis.^31^ Azathioprine is moderately effective in the treatment of non-infectious uveitis. There is evidence of systemic cyclosporine for treating intermediate and posterior uveitis which is comparable to systemic glucocorticoids.^30^

### Biologic and targeted synthetic DMARDs

Biologic DMARDs are recombinant DNA based therapies that consist of bioengineered soluble receptors, monoclonal antibodies, and cytokines that are responsible for expressing the pro and anti-inflammatory components of the immune system. An important pro-inflammatory cytokine TNF alpha is therapeutically very effective in treating several rheumatic diseases and their ocular manifestations. TNF antagonists including infliximab and adalimumab have been reported to reduce the frequency of AAU.^32–34^ A meta-analysis estimated the frequency of uveitis in patients with AS who were treated with infliximab and etanercept therapies and concluded that infliximab was more effective than etanercept.^35^ When Etanercept was indirectly compared to other monoclonal TNF antagonists, it showed higher AU flare rates. Several studies suggest that monoclonal antibodies are more effective than soluble receptors for the treatment of uveitis. Therefore, Etanercept is not recommended for treatment of spondyloarthritis with uveitis.^35^

Infliximab or adalimumab also may be used as glucocorticoids-sparing treatment for patients with chronic uveitis associated with SpA.^36^ Experience with other biologics in AAU such as Certolizumab and Golimumab is growing.^37,38^

## FUTURE PERSPECTIVES

Despite advanced therapies available for the treatment of uveitis, challenges persist. Although, there is an upsurge in the randomized controlled trials evaluating treatment interventions in SpA, there is a scarcity of data from head-to-head studies especially in the context of ocular manifestations. Such comparative data would provide better evidence required for clinical decision-making and healthcare economic assessments. Several biologic therapies have shown excellent results in patients with AAU associated with SpA. More evidence is needed to determine the efficacy of biologic therapy consisting of anti-TNF alpha in patients with AAU.

## CONCLUSION

**Figure 1.**
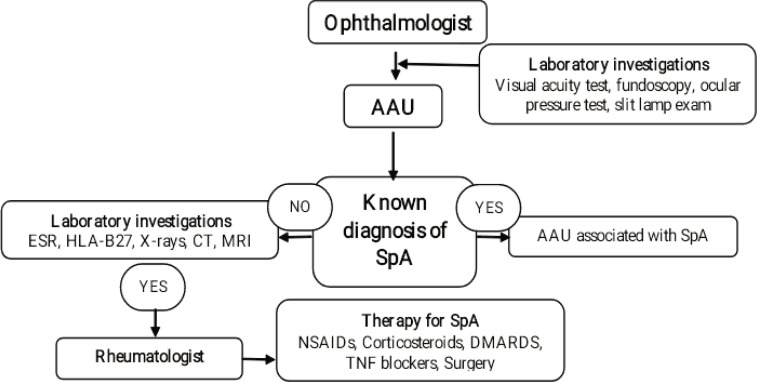
Management of uveitis in SpA. A collaborative approach of rheumatologist and ophthalmologist (based on Haroon M. et al.)^28^

Patients with SpAs may suffer from different ocular complications with uveitis being the most common one. Genes, gender, and geographical factors are the key factors impacting the epidemiological pattern of uveitis. Biological therapy is a promising medical strategy enabling us to achieve therapeutic goals accurately with minimal adverse effects. An effective management strategy for patients with AAU associated with SpA can be achieved by due collaboration between ophthalmologists and rheumatologists (**Figure 1**).^28^
